# A case of inflammatory pseudotumour of the gallbladder presenting as a big mass of uncertain behavior

**DOI:** 10.1186/s12876-020-01408-7

**Published:** 2020-08-24

**Authors:** Antonio Calvo, Jesús Salas, Gloria Muñoz, Ana Díez, María Coral de la Vega

**Affiliations:** Department of Surgery and Pathology, Puerto Real University Hospital, Cádiz, Spain

**Keywords:** Inflammatory Pseudotumor, Gallbladder

## Abstract

**Background:**

Inflammatory pseudotumour has been used to describe an inflammatory or fibrosing tumoral process of an undetermined cause that may involve a variety of organ systems, including the lungs, spleen, liver, lymph nodes, pancreas and extrahepatic bile duct with potential for recurrence and persistent local growth. In this article, we report a patient with a big mass of uncertain nature and behavior.

**Case presentation:**

A 60-year-old woman presented with a 1-week history of abdominal pain, fever and jaundice. Six months before she had had right upper quadrant pain that was interpreted as biliary colic. A contrast-enhanced CT scan showed a big mass of soft tissue with diffuse infiltration of the gallbladder, displacement of the transverse colon, hepatic flexure and duodenum. For diagnostic distinction between a chronic inflammatory disease or a neoplasm, exploratory laparotomy was required. Intraoperative exploration disclosed a big mass of hard texture involving the gallbladder, with multiple concrements, hepatoduodenal ligament, right and transverse mesocolon, stomach and duodenum.

Cholecystectomy was performed, preserving adjacent organs with macroscopic desmoplastic reaction. Histopathologic examination of the gallbladder showed a spindle cell proliferation with diffuse chronic inflammatory infiltrate of lymphocytes, plasma cells and hyalinized fibrous stroma. No vascular invasion or cellular atypia were evident.

**Conclusion:**

Inflammatory pseudotumour is a rare condition and diagnostic distinction from a chronic inflammatory disease or other neoplasm is only possible by histopathologic examination. There is a limited number of case reports in the literature indicating tumor location in the gallbladder.

## Background

Inflammatory pseudotumour is a rare lesion that has been described in various organs and tissues. Intra-abdominal variants of the disease are reported to occur most frequently in the liver, spleen, mesentery, and extrahepatic bile duct. The location of the gallbladder is even more uncommon.

Malignant transformations and recurrences of inflammatory pseudotumour have been reported years after surgery, therefore long-term follow-up is necessary even for patients successfully treated by surgical resection.

Elevated IgG4 serum levels have been reported in association with this illness as well as abundant IgG4 positivity in tumor infiltrating plasma cells, signs suggestive of an IgG4-related disease. A high serum IgG4 concentrations, thus provides a useful means of distinguishing this disorder from other differential diagnoses.

Pharmacologic treatments have also been reported for IgG4-associated inflammatory pseudotumor and there are even cases of complete resolution of the disease with steroids treatments. However in the presented case, this condition did not occur, so treatment was exclusively surgical.

## Case presentation

A 60-year-old woman presented to the Emergency Room with abdominal pain, fever, pruritus and jaundice since 1 week. The patient had a history of smoking and a family history of pancreatic cancer.

On physical examination, a hard and painful mass was identified on the right hypochondrium. Blood laboratory examination showed extrahepatic cholestasis enzymes (total bilirubin 8,44 mg/dl, direct bilirubin 8,31 mg/dl, AST 529 U/L; ALT 728 U/L; GGT 3974 U/L; LDH 353 U/L, alkaline phosphatase 876,4 U/L. In anamnesis, the patient referred to abdominal pain occurring during the last 6 months located in the right upper quadrant, which had been interpreted as biliary colic by her general practitioner.

Tumour markers and blood count showed no alterations. Viral serology, autoimmunity antibodies, metanephrines and urine normetanephrine were within the normal range.

**Fig. 1:**
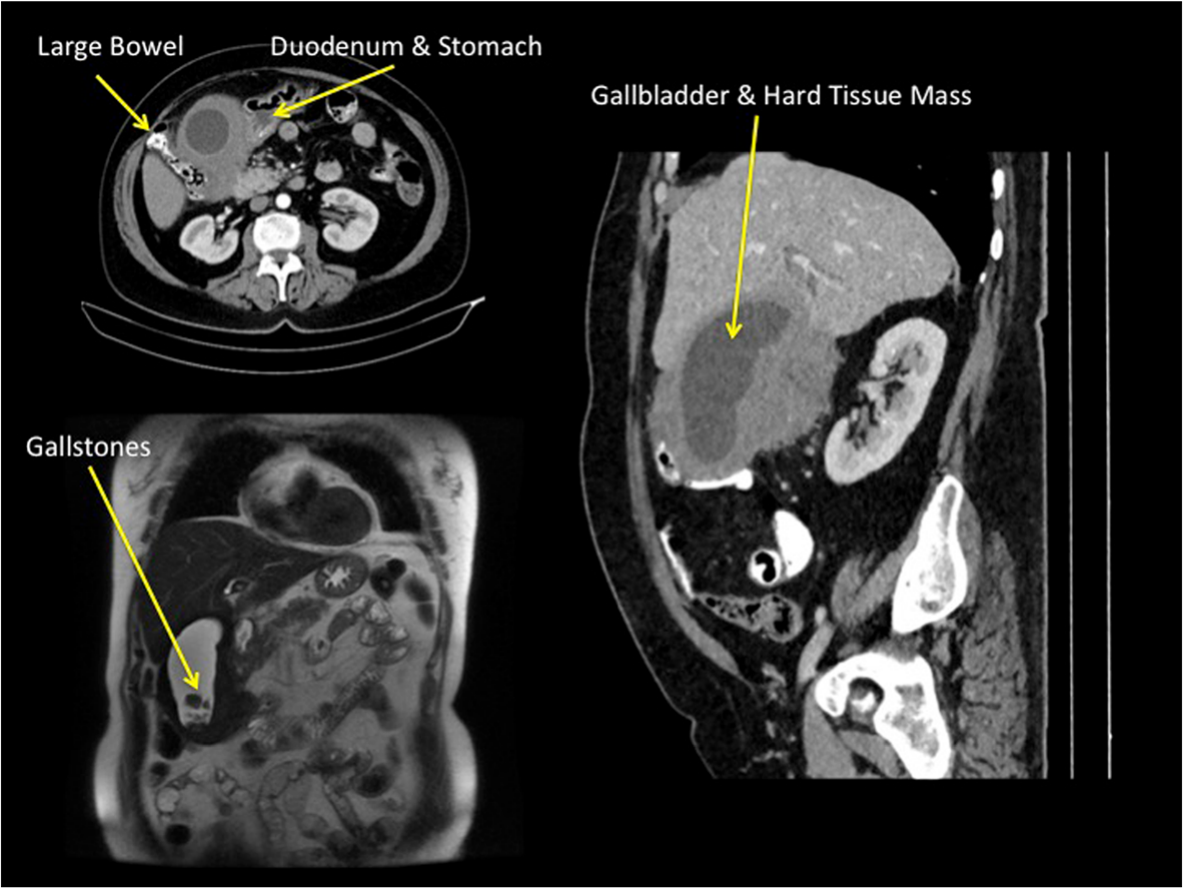
Axial and coronal computed tomography images showing a large mass of diffuse soft tissues originating from the gallbladder, and displacing the duodenum, transverse colon and hepatic flexure (**a**, **b**). In magnetic resonance imaging the gallbladder was distended and contained stones, associated with a slight dilatation of the intrahepatic bile duct (**c**)

A large mass associated with the gallbladder was identified by abdominal ultrasound. Contrast-enhanced CT scan disclosed a large soft tissue mass originating from the gallblader with homogenous contrast enhancement and without clear infiltration of the hepatic parenchyma. The mass displaced the transverse colon, hepatic flexure and duodenum. No lymphadenopathies were identified in the hepatoduodenal ligament, pancreas, retroduodenum or celiac axis (Fig. 1a, b) The gallbladder was distended, contained stones, and had a regular lumen, while there was slight dilatation of the intrahepatic bile duct on magnetic resonance imaging (Fig. 1c).

With these findings, a diagnostic distinction between a chronic inflammatory disease or a neoplastic process was necessary. The biopsy of the mass was performed under ultrasonographic control. Histopathologic examination showed spindle cells and some inflammatory cells of smaller size and absence of xanthic cells. The tumor showed a mesenchymal aspect that was confirmed by absence of epithelial cells on Pan-Cytokeratin staining, while some histiocytes were recognized. In summary, the pathology diagnosis was a mesenchymal process that could be reactive or malignant. The microbiological study of the bile obtained from gallbladder punctured showed a non-purulent Gram’s stain and negative cultures for both, aerobic and anaerobic germs. The oral endoscopy and biopsies of the second part of the duodenum didn’t show any pathological condition.

**Fig. 2:**
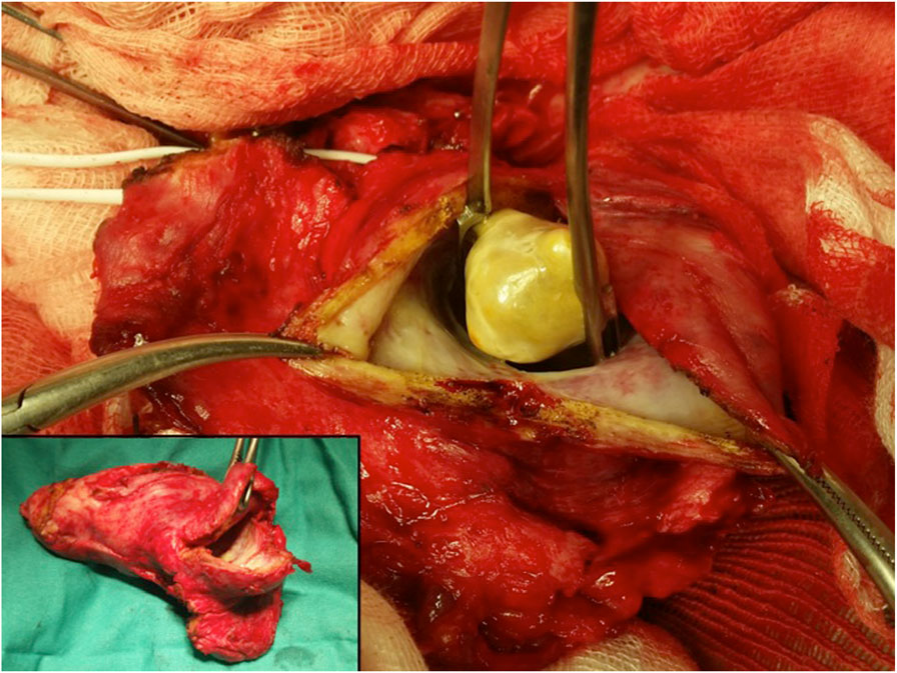
On laparotomy a large and hard mass was identified in the gallbladder with stones, displacing but not infiltrating right and transverse mesocolon, stomach, duodenum and hepatoduodenal ligament.

Exploratory laparotomy was decided and cholecystectomy could be performed, preserving the adjacent organs with macroscopic desmoplastic reaction. The mass was peeled off the transverse colon, first and the second part of the duodenum and common bile duct (Fig. 2).

The histopathological examination of the resection specimen disclosed sclerosing fibrous tissue with histiocytes, chronic lymphocytic inflammatory infiltrate and plasma cells with isolated eosinophils, and no epithelial malignancy. The mass presented as an expansive growth from the outer portion of the muscular layer of the gallbladder to the surrounding fatty tissue.

**Fig. 3:**
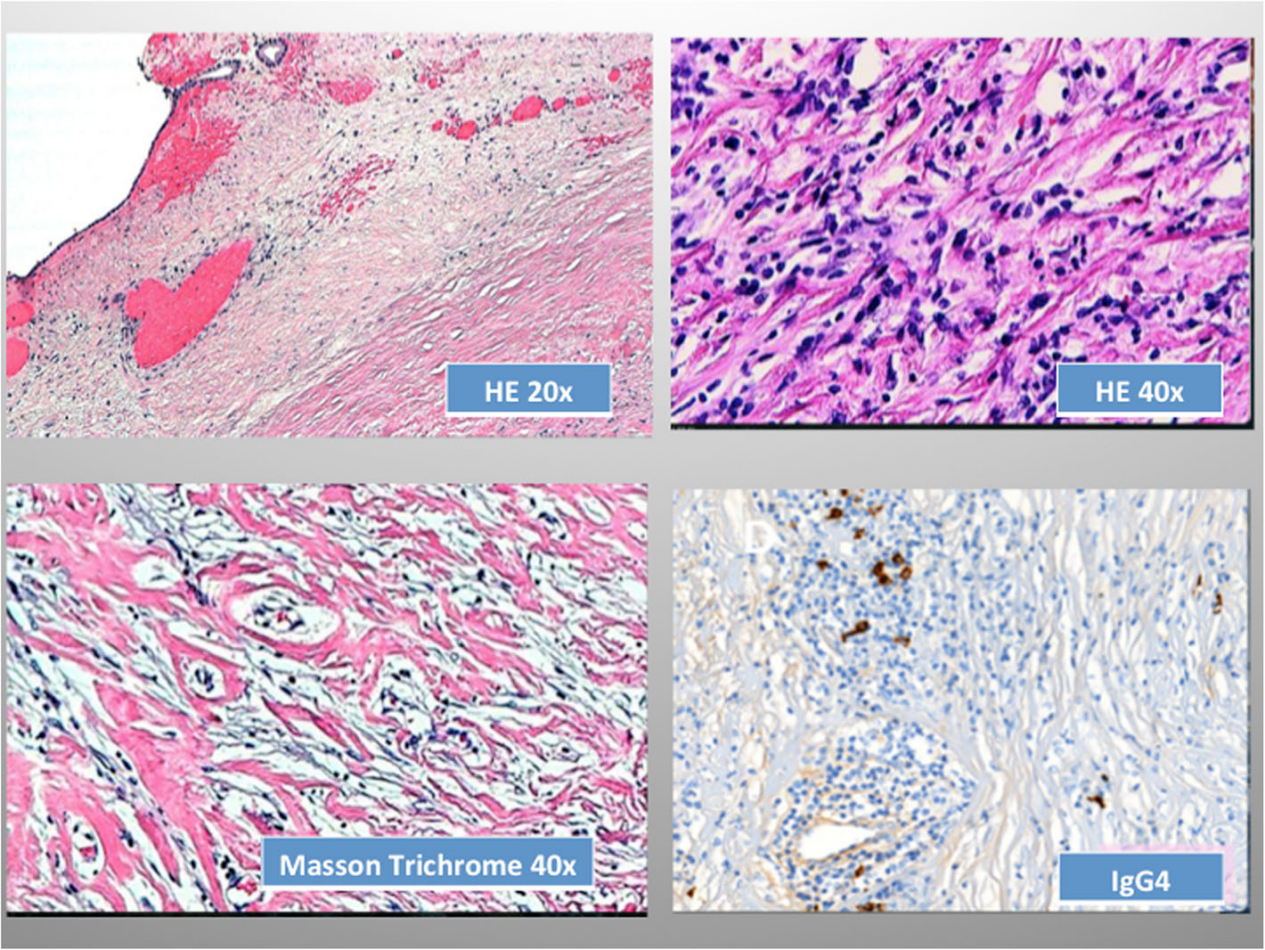
Histopathologic examination disclosed a thickened gallbladder wall (**a**) with spindle cells and proliferation of connective fibrous tissue without signs of celular atypia (**b**) and inflammatory cells, including lymphocites, plasma cells and hyalinized fibrous tissue, without vascular invasion (**c**). Few plasma cells were IgG4 positive in relation to the whole inflammatory cell infiltrate (**d**)

The definitive pathological diagnosis was inflammatory pseudotumour of the gallbladder with chronic sclerosing cholangitis (Fig. 3 a, b).

To achieve a definitive classification, complementary immunohistochemical stains were performed, and showed positive staining for smooth muscle actin in the muscular layer of the gallbladder and vessel walls; CD34+ in the vascular lumen; CD68+ in histiocytes, and remained negative for anaplastic lymphoma kinase (ALK) and Pan-Cytokeratin (PAN-CK). Masson’s trichrome stain showed intense positivity on collagen fibers. Less than 10% of the tumor cells sample were Ki67 positive, and 11 plasma cells were IgG4 positive per high power field. Taken together these findings confirmed the diagnosis of inflammatory pseudotumor of the gallbladder with sclerosing cholangitis associated with a normal level of serum of Immunoglobulin G4 of 24 mg/dl (10–67 mg/dL).

No local recurrence was detected at the three-years follow-up on CT scan.

## Discussion and conclusions

The term inflammatory pseudotumour has been used to describe an inflammatory or fibrosing tumoural process of undetermined cause that may involve a variety of organ systems, including the lungs, spleen, liver, lymph nodes, pancreas and extrahepatic bile duct with potential for recurrence and persistent local growth [1, 2]. There is a limited number of case reports in the literature indicating gallbladder location [3, 4].

Inflammatory pseudotumour appear to be more common in non-European populations. They usually occur in infancy and young adults but can occur in the elderly [5]. The IgG4 serum level should be determined due to a common association of elevated serum IgG4 with this illness, and some authors describe abundant IgG4 positivity in plasma cells as suggestive of IgG4-related disease [6].

High serum IgG4 concentrations might provide a useful means of distinguishing this disorder from other lesions [7–9].

The histopathological examinations showed sclerosing fibrous tissue with histiocytes, chronic lymphocytic inflammatory infiltrate and plasma cells with isolated eosinophils compatible with IgG4-related disease. However as IgG4 serum levels were within normal range and the IgG4 tissue expression was very weak, there is no strong evidence of a clear association with IgG4-related disease.

Inflammatory pseudotumour can generally be considered to be a relatively rare disease of undefined origin, with a great variety of symptoms, causing diagnostic challenges in the distinction of chronic inflammatory disease and neoplasm.

Inflammatory pseudotumor is defined as non-neoplastic but is currently considered as a tumour with low-grade malignant transformations. It has been reported in the liver, urinary bladder, kidney, breast, stomach, pancreas, spleen and retroperitoneum. There is also a limited number of case reports in the literature indicating the gallbladder location. These patients must be observed with close and regular long-term follow-up as recurrences have been reported to occur four to 7 years after surgery [10].

## Data Availability

Data sharing is not applicable to this article as no data sets were generated or analysed during the current study.
